# Infant temperament as a predictor of maternal attachment: A Jordanian study

**DOI:** 10.1002/nop2.668

**Published:** 2020-10-23

**Authors:** Sawsan Abuhammad, Manar AlAzzam, Rana AbuFarha

**Affiliations:** ^1^ Faculty of Nursing Jordan University of Science and Technology Irbid Jordan; ^2^ Princess Salma Faculty of Nursing Al‐Albayt University Mafraq Jordan; ^3^ Applied Science Private University Amman Jordan

**Keywords:** infant temperament, Jordan, maternal attachment, nurses, nursing, secure attachment

## Abstract

**Aim:**

To investigate the relationship between maternal bonding and infant temperament during the first year of infant's life. Moreover, it also wanted to explore which perinatal factors could influence the quality of maternal attachment.

**Method:**

A cross‐sectional study design was used to collect data from a sample of 277 mothers during the period of May–October 2018. The participants were asked to complete a three‐part survey that was developed to collect data on maternal attachment and infant temperament. Data was collected between March 2019–June 2019.

**Results:**

The analysis of the data revealed that there was a significant relationship between infant temperament and maternal attachment [*F*(2, 95) = 6.783, *p*‐value = .001]. It also revealed that the factors that most influenced maternal attachment were infant temperament and planned pregnancy, which together explained 54% of the variance in maternal attachment (*R* ^2 ^ =  7.5).

**Conclusion:**

Maternal attachment in Jordanian mothers can be explained by two significant factors: infant temperament and planning for pregnancy.

## 1 INTRODUCTION

The emotional bond that causes a mother to care for her infant and to have a feeling of commitment to her infant is called maternal attachment. This attachment and the quality of the interaction between mother and infant is a determinant of the social and cognitive development and potentially the mental health of the infant in later life (Abuhammad, 2016; Kim et al., 2017). An infant's characteristics, especially his/her temperament, play an important role in the quality of those interactions (Jonas et al., 2015; Tester‐Jones et al., 2015). This is because infant temperament and maternal attachment are very strongly connected and affect the nature and the quality of interaction (Abuhammad, 2016). It has been suggested that this interaction could be improved through some interventions, where the aim of such interventions would be to help to improve maternal attachment and the mother–infant relationship in general (Jonas et al., 2015; Kivijärvi et al., 2004). When infants are born, they carry constitutional characteristics (temperament) that encompass activity, emotional attachment, self‐regulation and reactivity (Solmeyer & Feinberg, 2011). Temperament is also affected by hereditary factors and by the environmental factors that exist around the infant during his/her daily life (Abuhammad, Khraisat, Joseph, & Al Khawaldeh 2020). Environmental, demographic and perinatal factors may contribute to inconsistencies among the studies, as several studies have revealed differences in socioeconomic, cultural and family backgrounds between preterm infants and their controls (Cho et al., 2016; Korja et al., 2010; Wolke et al., 2014). Furthermore, more immature preterm infants surviving today exhibit specific interaction characteristics that may affect the results of new studies on the mother–preterm infant attachment. Good‐quality maternal and infant attachment enhances the infant's later socio‐emotional, behavioural and cognitive development and is even related to the physical health of the child (Abuhammad et al., 2020). The infant needs to experience reciprocal affective interaction with the parent to become interested in social interactions and to develop secure attitude and positive behaviour towards mother and environment (Ren & Zhang, 2018; Solmeyer & Feinberg, 2011).

The centre of an infant's world is largely the primary caregiver(s), especially the mother and it is this world that shapes an infant's behaviour and how he/she functions in his/her world (Abuhammad, 2020a, 2020b; Ren & Zhang, 2018; Shin & Kim, 2007). However, the interaction between mother and infant is bidirectional, that is they affect each other (Abuhammad & Johnson, 2018; Leerkes & Zhou, 2018). The infant's reactions affect how the mother interacts with her infant. The infant's presentation and reactions are, at least in part, temperamentally based (Abuhammad et al., 2020). However, the mother's behaviour towards her infant is based on a mix of factors that include not only infant temperament, but also the mother's own level of attachment, behavioural control and satisfaction with her role as a mother (Hatamleh et al., 2018), as well as other life circumstances such as support and working conditions (Abuhammad, 2016).

Many studies have found that infant characteristics persist into later life (Kivijärvi et al., 2004; Solmeyer & Feinberg, 2011). Bowlby (1988), one of the pioneers of attachment theory, suggests that attachment patterns represent the quality of the dyadic relationship between mother and child. The attachment model is constructed during the first year after childbirth and is based on the interactions between the parents and their child. Therefore, when evaluating the attachment relationship, researchers need to consider the various aspects of temperament such as the infant's adaptation to new situations and accommodation to daily rituals such as sleep and mealtimes. Infants who have problems in adaptability and persistence may be considered as a source of parenting stress, which thus impairs the quality of attachment. An infant who receives sensitive and responsive care from his/her mother is able to consider him/herself as a person worthy of love and care and can depend on his/her mother at times of emotional need (Bretherton & Munholland, 2008). Thus, it might be anticipated that infant temperament could influence maternal attachment during the first year of a child's life.

However, only two studies have specifically considered the relationship between infant temperament and early maternal attachment and the maternal–infant relationship in general. In one of these studies, Parfitt et al. (2014) found that a difficult infant temperament is associated with a lower quality of maternal attachment and mother–infant relationship at 3 months after controlling for the statistical impact of the mental health of the mother. Similarly, the investigators found that at 15 months, a difficult infant temperament is associated with a lower quality of maternal attachment and the whole relationship between mother and her infant. In the only other study that specifically considered attachment, Edhborg et al. (2005) found that a difficult infant temperament is related to a lower quality of maternal attachment.

Furthermore, most studies on infant temperament and maternal attachment have been conducted in developed countries. In contrast, there is a lack of studies in developing countries such as Jordan. Hence, it is envisaged that this study will fill this gap in the literature as its main aim to investigate the relationship between maternal bonding and infant temperament during the first year of infant's life. Moreover, it also wanted to explore which perinatal factors could influence the quality of maternal attachment. The hypotheses were:

Hypothesis 1 Mothers who have an easy infant will demonstrate a greater attachment to their infants than mothers who have an unpredictable or difficult infant, as measured by the MAI.

Hypothesis 2 Perinatal factors could influence the quality of maternal attachment.

## 2 METHODS

### 2.1 Sample and data collection procedure

A cross‐sectional descriptive design was employed in this study. Women were recruited from three Maternal and Child Health (MCH) centres, each of which is located in a different neighbourhood of the city of Irbid in Jordan. Approximately 1.5 million people live in Irbid. Each MCH centre serves around 30,000 Jordanian women and their children and provides services that include prenatal care, postpartum care, family planning and immunization (Department of Statistics, 2014; Ministry of Health, 2010). A sample of Jordanian mothers was chosen for this study because Jordan is a developing Middle East Arabic country, which means that the results of this study could be generalized to other similar developing countries, especially those that located in the Middle East and thus the results could be of wider benefit to paediatric healthcare providers and mothers in several countries.

The inclusion criteria for this study were as follows: dyads consisting of Jordanian women aged more than 18 years with a full‐term infant 2–12 months in age. The mothers also had to be able to read and write in the Arabic language and must have previously visited one of the three MCH centres for immunization and infant care services. Only Jordanian women were included in this study. Syrian refugee women were excluded because war may have had a negative impact and their responses may therefore have been affected by other distinct factors that may have influenced their psychological status, which could have distorted the results of this study. Women with an infant who had experienced a serious illness such as a metabolic disease or cancer were also excluded from this study. Women were also excluded if they had stopped or did not initiate breastfeeding for medical reasons.

The researcher used a survey to collect data from Jordanian mothers after getting approval to conduct the study from the Deanship of Research of the Jordan University of Science and Technology (#2017232) and the Ministry of Health (#MOH REC170132). At each MCH centre, the researcher explained the purpose of the study to a research assistant who assisted in the recruitment of eligible women. The researcher asked the eligible women to complete the survey. The survey sent to 300 mothers and the mothers accepted to participate were 278. The women who rejected to participate excused for not having time to fill the survey. The research assistant explained the purpose of the study to the eligible women the details of which were also included in the cover letter attached to each survey form. The mothers were required to complete the survey in approximately 30 min. After completion, the participants deposited the completed questionnaire in a special collection box. The data were collected between May and October 2018.

### 2.2 Measures

The survey consisted of three parts. The first part consisted of three sections, each containing 10 questions designed to collect socio‐demographic data, current antenatal data and past breastfeeding experiences. The demographic data section included questions about maternal age, employment, marital status, educational status, number of children and the family's annual income and size. The antenatal section included questions about the mother's total number of pregnancies and the most recent type of delivery. The third section included questions on current feeding methods and a related question designed to elicit why the mother had decided to use her chosen method. As mentioned above, if the mother answered that she was not breastfeeding for a medical reason, she was excluded from the study.

### 2.3 Maternal attachment inventory

The second part of the survey was a self‐report form entitled the Maternal Attachment Inventory (MAI). This MAI form, which was initially developed by Müller (1994) and adapted for this study, consists of 26 items that measure the level of maternal attachment to infant cues. Specifically, the MAI measures the woman's recognition of her attachment to her infant and any perceived obstacles to expressing her attachment or responding to the attachment expressed by her infant to her infant. The MAI form covers the following three dimensions of maternal attachment: pleasure of proximity, tolerance and acceptance. The maternal response to infant behaviours is categorized into six categories: smile, eye contact, touch, auditory signal, eye directions and offering food. The infant behaviours include gazing towards or away from the mother as well as facial expressions in response to the mother's gaze (Gharaibeh & Halman, 2012). Cronbach's alpha value of MAI in this study was 0.85.

The frequency of the six maternal responses/signals for each of these three infant categories of behaviour is determined by the responses to the 26 items in the survey that are made according to a four‐point Likert scale, which ranges from almost never (1) to almost always (4). Hence, the total score can range from 26–104 points. In this study, in line with Shin et al. (2006), a score greater than 75 was considered to indicate a high level of maternal attachment. The MAI had already been validated for the current context by previous studies, which indicated that for the internal consistency reliability achieved a Cronbach's alpha value of 0.82 (Gharaibeh & Halman, 2012; Shin & Kim, 2007).

### 2.4 Infant characteristics questionnaire

The third part of the survey was the Infant Characteristics Questionnaire (ICQ). The original version of this instrument (Bates et al., 1979), which was developed to measure infant temperament based on parental perceptions, was adapted slightly for the current study. Firstly, a five‐point rather than a seven‐point Likert scale was used to gather the responses because the experts who helped in checking the reliability and validity of the instrument after it had been translated into the Arabic language suggested reducing the scale from seven to five to limit the number of responses that the subjects had to choose from and thus make it easier to complete. They also suggested using imperative sentences because in Arabic writing imperative rather than interrogative questions are more commonly used and thus more understandable for an Arabic‐language‐speaking sample.

The ICQ contains 20 questions designed to assess the easiness of the infant, based on its predictability, adaptation to the environment, attention‐seeking and emotionality. By way of example, the questions included: “Does your baby require things other than care‐giving?”, “What is your estimation of the activity of your infant in general?” and “How easy is it to take your baby places?” Based on the adapted five‐point Likert scale, the total infant temperament score could range from 20–100. Based on the total scores, the infants were divided into three categories: easy infant (20–40), unpredictable (40–60) and difficult (60–100). Cronbach's alpha values were good; the first's factor alpha is (0.86) and the Cronbach alpha for other factors were (0.78, 0.77, 0.46 and 0.59; Abuhammad et al., 2020).

### 2.5 Ethical approval

We sought the moral justification to conduct this study from the ethical committee Jordan University of Science and Technology (JUST) and the Health Ministry IRB. The researcher informed the participants about the purpose of the research, its benefits, freedom to participate and the freedom of one to recues them from the study without punishment. Besides, the subjects were assured of the confidentiality of their information and that only the researcher could access their information.

### 2.6 Data analysis

The collected data were entered into the Statistical Package for the Social Sciences version 25. The data were coded for statistical analysis only and contained no identifiers such as names, so there was no way to link the information obtained to individual participants. Continuous variables such as age and income were described using mean and standard deviation. Categorical variables such as education were described using frequency, frequency distribution and percentage (Rhodes et al., 2003). One‐way analysis of variance (ANOVA) statistics were used to compare the dependent variable (maternal attachment) between groups, the dependent variable, maternal attachment, was a scale level of measurement that is in congruence with the assumptions of ANOVA (Kong et al., 2012; Rhodes et al., 2003). There were two levels for each independent variable (interval and scale), as shown in Table 1 in the “Results” section. Maternal attachment scores between groups having low or high temperament (Kong et al., 2012; Rutherford & Mayes,  2011). In addition, hierarchical regressions were used to detect the factors that had an impact on maternal attachment.

**Table 1:** Characteristics of the study participants (*N* = 277)

Parameters	*N* (%)	Mean (*SD*)
Mother age (years)		27.8 (5.9)
Educational level		
Illiterate	18 (6.5)	
Primary	41 (14.8)	
Secondary	97 (35.0)	
Bachelor or more	121 (43.7)	
Employment status		
Employed	37 (13.4)	
Not employed	240 (86.6)	
Assistance in infant care		
Yes	107 (34.9)	
No	179 (65.1)	
Assistance is provided by		
Child grand parents	70 (65.4)	
Husband	30 (28.0)	
Others	7 (6.5)	
Experience in infant care		
Yes	206 (74.4)	
No	71 (25.6)	
Living in Independent house		
Yes	219 (79.1)	
No	58 (20.9)	
Perception towards marital relationship after childbirth		
Very good	223 (80.5)	
Good	37 (13.4)	
Fair	11 (4.0)	
Bad	6 (2.2)	
Family income level (Jordanian dinar)		399.1 (182.0)
Infant age (months)		8.5 (3.6)
Planning of Pregnancy		
Yes	193 (69.7)	
No	84 (30.3)	
Mode of delivery		
Normal Vaginal Delivery	184 (66.4)	
Cesarean section	93 (33.6)	
Received prenatal information about the infant care		
Yes	232 (83.8)	
No	45 (16.2)	
Source of information		
Health centre	118 (57.3)	
Mass media	30 (14.6)	
Family and friends	14 (6.8)	
Others	44 (21.4)	
Type of infant feeding		
Breast feeding	143 (51.6)	
Bottle feeding	56 (20.2)	
Together	78 (28.2)	
Perceived experience during pregnancy and childbirth		
Very good	126 (45.5)	
Good	71 (40.8)	
Fair	27 (9.7)	
Bad	10 (3.6)	

Note

One Jordanian dinar is equal to 1.4 US dollars.

## 3 RESULTS

### 3.1 Demographic data

The age of the mothers ranged from 18–40 years, and the mean age was 27.3 years. Approximately 61% (*N* = 150) of the mothers had not completed a diploma level of education. Most of the mothers reported that they were unemployed (86.6%; *N* = 240). The mean family monthly income was 339.5 Jordanian dinars (780 US dollars). About one‐fifth of the women were living with an extended family (20.9%, *N* = 58). As regards the demographic characteristics of the infants, their ages ranged from 2–12 months and the mean age was 8 months. Almost 46.3% (*N* = 129) of the infants were female, and 53.7% (*N* = 148) were male. Approximately 36% (*N* = 94) of the infants were in families who received assistance with caring for their infants. Mothers who received assistance 65% (*N* = 70) received that assistance from the grandparents of their child. Most of the mothers reported having previous experience of caring for an infant 74.4% (*N* = 206). Most mothers reported that the relationship with their husband was very good 80.5% (*N = *223), whereas 2.2% (*N = *6) reported that they had a poor relationship with their husband.

Approximately 66.4% (*N* = 193) of the mothers had planned their pregnancy, 66.4% (*N* = 193) had had a vaginal delivery and 51.6% (*N* = 143) were breastfeeding at the time of the study. Most mothers had received antenatal education and care during pregnancy (83.8%; *N* = 232). Around half of the participants described their pregnancy as very good. Table 1 presents the full results of the descriptive analysis of the demographic and perinatal characteristics of the participants and their infants.

### 3.2 MAI and ICQ data

The data collected via the MAI and ICQ were used to test the following two hypotheses:

Hypothesis 1 Mothers who have an easy infant will demonstrate a greater attachment to their infants than mothers who have an unpredictable or difficult infant, as measured by the MAI.

Hypothesis 2 Perinatal factors could influence the quality of maternal attachment.

**Figure 1:**
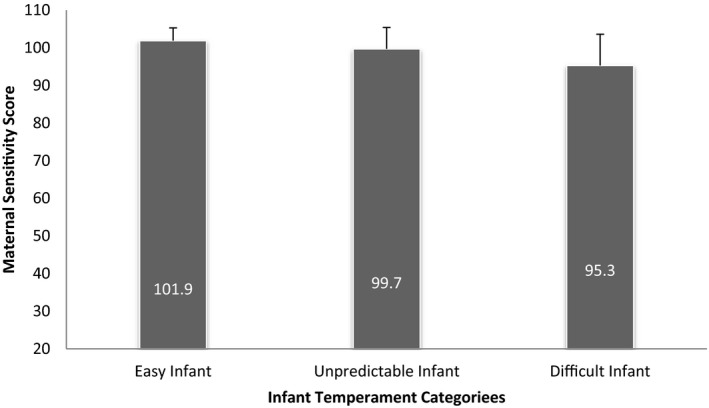
The difference in maternal attachment score between mothers based on the child temperament category. *Note:* The test was conducted using ANOVA test (*F*(2, 95) = 6.783, *p* value = .001). *The numeric values imbedded in the bar graphs reflecting maternal attachment score at each type of infant temperament

The first hypothesis was examined by using ANOVA, and the results revealed significant differences in maternal attachment between mothers according to the infant temperament score. The maternal attachment score for mothers with an easy infant was significantly greater than that for mothers with an unpredictable and difficult infant (*p* = .004 for both). Also, mothers with an unpredictable infant showed a higher maternal attachment score compared with mothers with a difficult infant (*p* = .05). See Figure 1.

To test hypothesis 2, hierarchical regression analysis was performed to determine the relative contribution of variables in predicting positive maternal attachment. As shown in Table 2, infant temperament, income, education, employment, assistance, who assist, experience, housing and marital status were entered into the model first as a control variable, accounted for 0.54% to the variance in positive psychological attitude (*p* < .001). At Step 2, income, education, employment, assistance, who assist, experience, housing, marital status, pregnancy planned or not, delivery method, receive prenatal data, source of data, feeding method. This accounted for 0.65% to the variance in positive attachment (*p* < .001). Step 3 of the model showed that infant variables are not significant and have an impact on the model (Table 2).

**Table 2:** Hericheral regression for the predicators of maternal attachment for infant

Model		Unstandardized coefficients		Standardized coefficients	*t*	Sig.
		*B*	*SE*	Beta		
1	(Constant)	30.245	4.501		6.720	0.000
	SUMB	0.198	0.048	0.417	4.137	0.000
	Income	−0.061	0.079	−0.088	−0.772	0.442
	Education	−0.888	0.503	−0.187	−1.765	0.082
	Employment	−0.308	1.150	−0.029	−0.268	0.790
	Assistance	−3.070	1.325	−0.234	−2.318	0.023
	Who assist	1.140	0.605	0.187	1.885	0.063
	Experience	−0.220	1.203	−0.020	−0.183	0.855
	Housing	0.454	1.169	0.041	0.388	0.699
	Martial status	−1.671	0.693	−0.245	−2.413	0.018
2	(Constant)	36.599	5.880		6.224	0.000
	Temperament	0.171	0.046	0.361	3.708	0.000
	Income	−0.089	0.082	−0.127	−1.083	0.283
	Education	−0.777	0.487	−0.164	−1.594	0.115
	Employment	−1.207	1.179	−0.115	−1.024	0.309
	Assistance	−2.651	1.261	−0.202	−2.103	0.039
	Who assist	1.298	0.586	0.213	2.215	0.030
	Experience	−0.492	1.150	−0.044	−0.427	0.670
	Housing	0.468	1.136	0.042	0.412	0.682
	Marital status	−2.251	0.690	−0.330	−3.263	0.002
	Pregnancy planned or not	2.640	1.120	0.252	2.358	0.021
	Delivery method	−0.951	1.042	−0.096	−0.913	0.365
	Receive prenatal data	−5.073	4.326	−0.116	−1.172	0.245
	Source of data	0.710	0.414	0.176	1.714	0.091
	Feeding method	−0.719	0.593	−0.132	−1.213	0.229
3	(Constant)	35.600	6.034		5.900	0.000
	SUMB	0.158	0.049	0.333	3.230	0.002
	Income	−0.082	0.083	−0.118	−0.992	0.325
	Education	−0.730	0.498	−0.154	−1.465	0.148
	Employment	−1.230	1.224	−0.117	−1.005	0.318
	Assistance	−2.939	1.305	−0.224	−2.252	0.027
	Who assist	1.231	0.599	0.202	2.055	0.044
	Experience	−0.594	1.163	−0.053	−0.510	0.611
	Housing	0.577	1.151	0.052	0.502	0.618
	Martial status	−2.100	0.721	−0.308	−2.913	0.005
	Pregnancy planned or not	2.750	1.172	0.262	2.346	0.022
	Delivery method	−1.177	1.096	−0.118	−1.075	0.286
	Receive prenatal data	−4.733	4.381	−0.108	−1.080	0.284
	Source of data	0.713	0.418	0.177	1.706	0.093
	Feeding method	−0.716	0.601	−0.131	−1.191	0.238
	Infant age	−0.027	0.144	−0.020	−0.185	0.854
	Infant gender	0.955	1.022	0.101	0.935	0.353
a.						

Significant at 0.05 significance level.

## 4 DISCUSSION

Here, the results are discussed and analysed in the Jordanian cultural context, which places great importance on the mother. The results should be of interest to all healthcare providers involved in mother and infant issues, such as maternal attachment and maternal and child attachment. The level of maternal attachment reflects the strength of the emotional bond that causes a mother to care for her infant and have a feeling of commitment towards her child. In this study, Jordanian mothers had high scores for maternal attachment to their infants.

The findings of this study also showed that infant temperament was a predictor of maternal attachment to the infant, which is consistent with the literature (Abuhammad, 2016; Abuhammad & Johnson, 2018). This means that having an easy infant may cause the mother to be more attached and responsive to her infant's needs. Jonas et al. (2015) reported that infant temperament affects maternal attachment and that increased maternal attachment is mediated infant temperament (effect ab = 0.0312 [0.0208], 95% CI = 0.0884–0.0031). The investigators also found that when infant temperament is controlled for, breastfeeding mothers display greater maternal attachment than formula‐feeding mothers.

The current study also supports previous work that shows that having a difficult infant might cause the mother to be less attached (Abuhammad, 2016). Similarly, in another study, Stams et al. (2002) examined the correlation between infant temperament and maternal attachment in adopted children. The investigators found that a difficult temperament in adopted children also plays a key role in maternal attachment and could lead to the development of internalized behaviour problems in the child. Moreover, Leerkes and Zhou (2018), who examined the association between infant temperament and maternal attachment, found that infants who are high in negative emotionality (difficult temperament) may be more strongly influenced by maternal attachment because they are highly dependent on their mother for external support to help them feel safe or because they are distressed more often.

Furthermore, Strathearn et al. (2012) found that having an infant with a difficult temperament is correlated with lower maternal attachment and lower interaction between the mother and her infant. The study by Strathearn et al. also revealed that mothers who have infants with a difficult temperament used kissing more often and used less vocalization, which the investigators suggested may influence the flow and the quality of interaction and the self‐control of the infant. Moreover, they also found that mothers of difficult‐tempered infants play less with their infant compared with mothers who have easy infants. This decreases the type of play interaction that may help in building the cognitive ability of the infant during their lifespan (Abuhammad et al., 2020). Similarly, Nolvi et al. (2016) and Moe et al. (2018) found that having an infant with negative temperament was related to a lower quality of attachment between such infants and their parents and also had an adverse impact on the mothering of the infant.

As regards other factors that may have had an effect on maternal attachment, the analysis conducted in the current study indicated that, besides infant temperament, the only other factor that contributed to explaining the variance in maternal attachment was planning for pregnancy. One plausible explanation for this finding is that planning for a pregnancy improves maternal attachment by helping both the mother and the father to be sufficiently prepared to take on their parental responsibility and welcome a new family member. On the other hand, Bielawska‐Batorowicz and Siddiqui (2008), who investigated the factors affecting attachment among future Swedish and Polish mothers, found that planning for pregnancy has no effect on maternal attachment during pregnancy or postpartum to the infant. Similarly, Gharaibeh and Halman (2012), who investigated the factors affecting maternal attachment among first‐time mothers in Jordan, showed that planning for pregnancy does not have an effect on a mother's affection for her infant, unlike maternal self‐efficacy, marital relationship quality and delivery experience which were all shown to influence the level of attachment. The investigators explained these results by suggested that Jordanian couples want to become parents as soon as possible because they fear being infertile and if they find that they are, they want to get early access to therapeutic treatment. They also stated that the family of the wife usually wants her to have a baby early in the marriage to prevent her husband from entering into a second marriage. Damato (2004) found that planning of pregnancy is a major predicator of perinatal attachment that late impact postnatal maternal attachment and Ustunsoz, et al. (2010) after comparing prenatal attachment between two groups of women who planned of pregnancy and who not, they found that planned of pregnancy women exhibited more prenatal and postnatal attachment.

As regards the impact of breastfeeding, the current study found that this factor has an impact on maternal attachment. This finding supports Abuhammad et al. (2020) and Abuhammad (2020a, 2020b), who found that breastfeeding affects both infant temperament and maternal attachment. The current study also found the infant's age and gender did not result in any differences in the level of maternal attachment. This finding could be explained by the young age (2–12 months) of all the infant subjects in this study, as male and female characteristics are not particularly distinguishable at this stage (Al‐Akour, 2008). The finding of the current study is consistent with Abuhammad (2016), who examined the impact of breastfeeding and other factors on maternal attachment and found that the age and gender of the infant does not influence the level of maternal attachment to the infant.

## 5 LIMITATIONS

Despite the importance of the above findings on maternal attachment among mothers in Jordan, a developing country, it should be noted that some limitations need to be taken into account. The major limitation of this study is that it used a convenience sample. The main drawback of using a convenience sample is sampling bias, such that the results of the study may not represent the entire population (Abuhammad, 2020a, 2020b) and are therefore not generalizable to the whole population. This means that this study has low external validity. A further limitation of this study is that it focused on a fairly homogenous group of almost exclusively Caucasian, low to middle class, educated, Jordanian women. As such, the findings are not fully representative from a cultural perspective and are thus not more widely generalizable (Hamadneh et al., 2018). This is a particularly important limitation in a study on the effect of infant temperament on maternal attachment because we know that maternal attachment is highly influenced by cultural norms. In addition, this study was not designed to assess objective differences that used observation instead of subjective self‐report in maternal attachment.

## 6 IMPLICATIONS FOR PRACTICE AND/OR POLICY

Despite the limitations highlighted above, the study provides some useful findings that may assist nurses and healthcare providers who work with mothers and infants to better understand the extent to which certain factors affect maternal attachment to an infant and the level of attachment between mothers and their infants. Such an understanding will enable nurses and healthcare providers to empower mothers and assist them more appropriately.

## 7 CONCLUSION

Maternal attachment in Jordanian mothers was explained by two significant factors: infant temperament and planning for pregnancy. Infant temperament was the main factor that explained maternal attachment; having an easy infant had a strong impact on the ability of the mother to clearly understand her infant's needs in an accurate way and in a timely manner. However, the results also revealed the importance to mothers of planning their pregnancy as this helps them to psychologically accept responsibility for their new infant. Finally, as differences in the level of maternal attachment were evident among these Jordanian mothers, it is also crucial to enhance our understanding of the influence of culture on mothers.

## CONFLICT OF INTEREST

The authors have no funding or conflict of interest to disclose.

## AUTHOR CONTRIBUTIONS

SA, MA and RF: Study and design, analysis and data interpretation, and critical revision and drafting of the manuscript. SA and RF: Data acquisition.

## Data Availability

The data had sensitive information about the participants that could not sharing with none participating authors in this study.
